# Inhibition of constitutive activity of the atypical chemokine receptor 3 by the small-molecule inverse agonist VUF16840

**DOI:** 10.1016/j.molpha.2025.100085

**Published:** 2025-10-26

**Authors:** Reggie Bosma, Desislava Nesheva, Merel Rijnsburger, Rick Riemens, Justyna M. Adamska, Max Meyrath, Simon Mobach, C. Maurice Buzink, Suzanne van der Pol, Iwan J.P. de Esch, Martyna Szpakowska, Maikel Wijtmans, Andy Chevigne, Henry F. Vischer, Rob Leurs

**Affiliations:** 1 Department of Medicinal Chemistry, Amsterdam Institute for Molecular and Life Sciences (AIMMS), Faculty of Science, Vrije Universiteit Amsterdam, Amsterdam, The Netherlands; 2 MS Center Amsterdam, Department of Molecular Cell Biology and Immunology, Amsterdam Neuroscience, Amsterdam University Medical Center, Amsterdam, The Netherlands; 3 Immuno-Pharmacology and Interactomics, Department of Infection and Immunity, Luxembourg Institute of Health, Esch-sur-Alzette, Luxembourg

**Keywords:** Atypical chemokine receptor 3, Inverse agonist, Constitutive activity, CXCL12, CXCL11

## Abstract

The atypical chemokine receptor 3 (ACKR3) has emerged as a promising drug target for the treatment of cancer, cardiovascular, and autoimmune diseases. In this study, we present the pharmacological characterization of VUF16840, the first small-molecule inverse agonist of ACKR3. VUF16840 effectively displaces CXC chemokine ligand 12 binding to ACKR3 and inhibits chemokine-induced *β*-arrestin2 recruitment in a concentration-dependent manner. Furthermore, VUF16840 stabilizes the inactive conformation of ACKR3, as demonstrated by its ability to suppress constitutive recruitment of downstream effector proteins. This inverse agonism alters ACKR3 constitutive trafficking, leading to receptor enrichment at the plasma membrane and inhibition of intracellular CXC chemokine ligand 12 uptake. Importantly, VUF16840 exhibits high selectivity for ACKR3 over a broad panel of human chemokine receptors. These findings establish VUF16840 as a potent and selective ACKR3 inverse agonist capable of modulating constitutive and chemokine-induced signaling and internalization events. As such, VUF16840 represents a valuable pharmacological tool for exploring the molecular and translational roles of ACKR3 in both physiologic and pathologic contexts.

**Significance Statement:**

A small molecule inverse agonist of the atypical chemokine receptor 3 (ACKR3), named VUF16840, is characterized in this work. It was shown that VUF16840 was able to inhibit basal as well as ligand-induced ACKR3 activation and, moreover, inhibits the scavenging function of ACKR3.

## 1 Introduction

The atypical chemokine receptor 3 (ACKR3), formerly known as CXCR7, was initially identified as a second receptor for the CXC chemokine ligand 12 (CXCL12), previously thought to exert its effects solely via the CXC chemokine receptor 4 (CXCR4).^1^^,^^2^ Because its discovery, ACKR3 has been extensively investigated for its pathologic roles, including its contributions to tumor progression and antitumor therapy resistance.^3^ Additionally, ACKR3 has been implicated in cardiovascular diseases,^4^, ^5^, ^6^ HIV entry into human host cells^7^ and autoimmune disorders^3^ underscoring its potential as a viable therapeutic target.

ACKR3 is a member of the G protein-coupled receptor (GPCR) superfamily and binds the chemokines CXCL11 and CXCL12 with subnanomolar affinities.^1^^,^^2^ More recently, ACKR3 has been shown to bind a number of opioid peptides^8^ suggesting a novel role for ACKR3 in pain perception. Unlike typical GPCRs, ACKR3 does not activate G proteins upon ligand binding.^2^^,^^9^^,^^10^ However, ligand binding increases ACKR3 interaction with *β*-arrestins, a process commonly used as a functional readout for ACKR3 activation.^8^, ^9^, ^10^, ^11^, ^12^ The recruitment of these intracellular regulatory proteins upon GPCR activation translates to diverse cellular responses for numerous GPCRs.^13^^,^^14^ Upon chemokine binding, ACKR3 rapidly internalizes from the cell membrane, scavenging bound chemokines from the extracellular milieu for intracellular degradation.^1^^,^^10^^,^^12^ This scavenging activity modulates the extracellular levels of CXCL11 and CXCL12, which are critical for directional cell migration mediated by CXCR3 and CXCR4, respectively, thereby influencing, for example, cancer metastasis and immune cell migration in conditions such as multiple sclerosis (MS).^3^^,^^15^

The scavenging function of ACKR3 can be disrupted by ligands that inhibit chemokine binding, regardless of whether they are agonists or antagonists. Consequently, controversy has arisen regarding the classification of ACKR3 ligands. For instance, compounds such as CCX754, CCX662, CCX733, and CCX771, often described as inhibitors or antagonists of ACKR3,^15^, ^16^, ^17^, ^18^, ^19^ are capable of stimulating ACKR3-mediated *β*-arrestin2 recruitment.^12^^,^^16^^,^^20^ In fact, to date most small molecules reported in the literature or natural compounds such as conolidine^19^^,^^21^^,^^22^ induce *β*-arrestin recruitment to ACKR3. Accordingly, small molecule ACKR3 agonists have been widely used to probe its scavenging function, showing efficacy in various cancer models,^2^^,^^3^^,^^15^^,^^17^^,^^20^ whereas inhibition of ACKR3 activity remains underexplored.

**Fig. 1:**
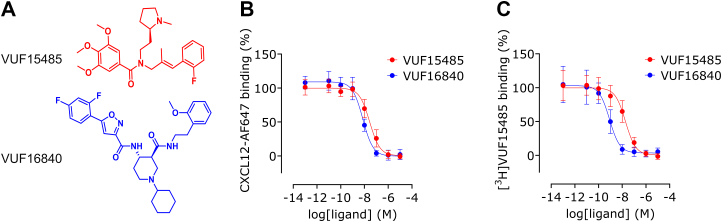
Concentration-dependent displacement of binding of labeled agonists to ACKR3. (A) Structures of displacers VUF15485 and VUF16840. HEK293T membranes transiently expressing either NLuc-hACKR3 or HA-hACKR3 were used to measure (B) the concentration-dependent displacement of the binding of fluorescently labeled CXCL12-AF647 or (C) the displacement of the binding of radioligand [^3^H]VUF15485 by unlabeled VUF15485 and VUF16840. Percentage of residual probe binding was normalized and the depicted data are the mean ± SD of n experiments. VUF15485 was evaluated in 17 experiments and 3 experiments and VUF16840 in 9 and 3 experiments in panel B and C, respectively with triplicate measurements per experiment.

Following up on previous work on small molecule ACKR3 agonists,^21^^,^^23^ we launched a research program aimed at identifying ACKR3 antagonists. At the start of this project, potent ACKR3 antagonists were not described in the scientific literature. We therefore performed an analysis of patent literature, as several companies had applied for patent protection of various chemical scaffolds acting on ACKR3, but often referred to those as CXCR7 or ACKR3 modulators.^19^^,^^21^^,^^24^ In this exploratory study, we identified a series of ligands described in patent application WO201819929 of Idorsia Pharmaceuticals^25^ and claimed to antagonize ACKR3. One of the isoxazole-based ligands, VUF16840 (Fig. 1A), was synthesized in-house and its detailed pharmacological characterization is described in this study. Notably, VUF16840 acts as an inverse agonist, suppressing constitutive signaling of ACKR3. This inverse agonism was observed in assays measuring the recruitment of *β*-arrestin1/2 or G protein-coupled receptor kinase 2 (GRK2), as well as in assays related to receptor internalization dynamics, leading to reduced constitutive endosomal translocation, enhanced cell surface localization and reduced cellular CXCL12 uptake. Interestingly, VUF16840 inhibition with ACKR3 is nonsurmountable by chemokines, resulting in potent inhibition even at high CXCL11 or CXCL12 concentrations. Moreover, VUF16840 demonstrates high selectivity for ACKR3 over other chemokine receptors, making it a valuable tool for investigating the role of ACKR3 constitutive activity and the therapeutic potential of ACKR3 antagonism in diverse biological contexts. During the course of our study, a close analog, ACT-1004-1239, was disclosed as an ACKR3 antagonist in the literature by Idorsia Pharmaceuticals^26^^,^^27^ and presented as a clinical candidate for the treatment of MS,^28^, ^29^, ^30^ making the present results relevant for the understanding of the potential therapeutic effect of ACKR3 modulation in MS.

## 2 Materials and methods

### 2.1 Materials

Hank's Balanced Salt Solution (HBSS) (with Ca^2+^ and Mg^2+^), Dulbecco’s modified Eagle’s medium (high glucose), 0.05% trypsin solution and penicillin/streptomycin solution was purchased from Thermo Scientific. Materials for radioligand binding, GF/C filter plates and MicroScint-O, were purchased from Perkin Elmer. FBS was obtained from Bodinco. Linear 25 kDa polyethylenimine (PEI) was purchased from Polysciences. Bovine serum albumin (BSA) was obtained from Melford. Furimazine (NanoGlo) and coelenterazine-h substrates were purchased from Promega. Resazurin is obtained from Merck. 96-Well plates were obtained from Greiner Bio-one. White low-volume 384 well plates were bought from Corning.

VUF15485 and VUF16840 were synthesized and characterized in-house (Supplemental Figs. 1–4). Human recombinant CXCL11 (#CN-13), CXCL12 (#CN-11), and fluorescently labeled CXCL12-A647 (#CAF-11) were purchased from Almac or Protein Foundry. All used chemicals were of analytical grade purity.

### 2.2 Plasmids

The NLuc-ACKR3 plasmid was a kind gift from Prof Hill (Nottingham University, Nottingham, United Kingdom),^31^ Venus-Rab5a was a kind gift from Dr Lambert (Georgia Health Sciences University, Augusta, GA, United States)^32^ and *β*-arrestin2-LgBiT/pBiT1.1-C[TK/LgBiT] and SmBiT-clathrinA/pBiT2.1-N[TK/SmBiT] plasmids were kindly provided by Dr Seong (Korea University, Seoul, Republic of Korea).^33^ The mVenus-CAAX and the hACKR3-NLuc constructs have been described by Zarca et al.^23^ The sequence of the used ACKR3 constructs corresponds to National Center for Biotechnology Information (NCBI) database entry NM_020311.3. Radioligand binding studies used a mammalian expression vector pcDEF_3_ encoding ACKR3, N-terminally fused to an HA-tag and C-terminally fused to a triple Alanine tag (NotI). This construct was the template for the C-terminal ACKR3 fusion with the luminescent Rluc8 protein (NCBI: EF446136.1). The CXCR4-Rluc8 fusion protein corresponds to CXCR4 isoform b (NCBI: NM_003467.2) fused at the C-terminus with a triple Alanine linker (NotI) before the Rluc8 protein.

For bioluminescence resonance energy transfer (BRET) experiments, several BRET acceptor constructs were used that consist of the pcDEF_3_ vector with either GRK2 (NCBI: NM_174710.2), *β*-arrestin1 (NCBI: NM_174243.3), or *β*-arrestin2 (NCBI: XM_005220181.4) fused at the C-terminus with a SpeI/NotI linker to mVenus (NCBI: KP666136.1), as previously described.^34^

For NanoBiT-complementation experiments, the HA-tagged ACKR3 was C-terminally fused to a SmBiT separated by a TSSGSSGGGGSGGGGSS-linker in pcDEF_3_, as previously described.^35^ The LgBiT-*β*-arrestin2 construct was generated by flanking the open reading frame of *β*-arrestin2 with SacI and XbaI restriction sites at the N- and C-termini, respectively, by polymerase chain reaction using the aforementioned *β*-arrestin2-mVenus as template. The resulting *β*-arrestin2 fragment was subcloned in the pBiT1.1-N[TK/LgBiT] vector (kindly provided by Promega) using the indicated restrictions enzymes. The NanoBiT constructs that were used for screening the pharmacological activity of VUF16840 on the panel of human chemokine receptors were described before.^8^

### 2.3 HEK293T cell culture

HEK293T cells (RRID: CVCL_0063) were maintained in Dulbecco’s modified Eagle’s medium, supplemented with 10% FBS and 1× penicillin/streptomycin. Cells were grown in 10 cm dishes and kept in a humidified atmosphere with 5% CO_2_ at a temperature of 37 °C. When cells reached a near confluent monolayer (∼85%), or in preparation of further experimentation, cells were briefly incubated with a 0.05% trypsin solution and subsequently collected in culture medium. Cell numbers were quantified using a TC20 cell counter from BioRad and subsequently seeded at the appropriate density for propagation or experimentation.

### 2.4 HEK293T cell transfection

For 1·10^6^ HEK293T cells, a transfection mix consisting of 2 *μ*g plasmid DNA, 12 *μ*g linear PEI in a total volume of 200 *μ*L 150 mM NaCl was made and thoroughly vortexed. The transfection mix was incubated for 15 minutes and consequently added to a cell suspension with a final concentration of 3·10^5^ cells/mL. For functional studies this cell suspension was immediately transferred to a poly-L-lysine-coated white 96-well plate, with 3·10^4^ cells per well. For BRET experiments, the 2 *μ*g plasmid DNA of the transfection mix consisted of 0.4 *μ*g Rluc8-fused chemokine receptors (ACKR3-Rluc8 or CXCR4-Rluc8) and 1.6 *μ*g of one of the mVenus-fusion proteins (*β*-arrestin1-mVenus, *β*-arrestin2-mVenus, GRK2-mVenus, and Rab5a-mVenus). For BRET-based membrane localization experiments, the 2 *μ*g plasmid DNA consisted of ACKR3-NL (0.05 *μ*g), mVenus-CAAX (0.25 *μ*g), and pcDEF3 vector DNA without insert (1.7 *μ*g). For NanoBiT-complementation experiments detecting *β*-arrestin2 recruitment to the ACKR3, the 2 *μ*g DNA of the transfection mix consisted of 0.4 *μ*g ACKR3-SmBiT, 0.6 *μ*g *β*-arrestin2-LgBiT, and 1 *μ*g pcDEF_3_. Finally, to detect the interaction between *β*-arrestin2 and clathrin A using NanoBiT, the 2 *μ*g DNA of the transfection mix consisted of 0.4 *μ*g ACKR3, 0.6 *μ*g *β*-arrestin2-LgBiT, 0.6 *μ*g SmBiT-Clathrin A, and 0.4 *μ*g pcDEF_3_.

### 2.5 Membrane preparations

Membranes of HEK293T cells that transiently expressed the NLuc-hACKR3 or HA-hACKR3 were produced as previously described.^23^ Briefly, 2 million HEK293T cells were seeded per 10 cm^2^ dish and were transfected the following day using a DNA/PEI-mix containing 0.25 *μ*g plasmid DNA encoding for the ACKR3, 4.75 *μ*g pcDEF3 plasmid DNA and 30 *μ*g linear PEI. Two days after transfection, cells were collected in PBS (137 mM NaCl, 2.57 mM KCl, 1.5 mM KH_2_PO_4_, 8 mM Na_2_HPO_4_) and pelleted by centrifugation. Cell pellets were resuspended in ice-cold membrane buffer (15 mM Tris, 0.3 mM EDTA, 2 mM MgCl_2_, pH 7.4 at 4 °C) and dounce-homogenized on ice by plunging the pestle 10 times with 1500 rpm. Cell homogenates were subsequently subjected to 2 freeze-thaw cycles using liquid nitrogen. Next, the cell homogenates were centrifuged at 40,000 *g* and pellets were resuspended in Tris-sucrose buffer (20 mM Tris, 250 mM sucrose, pH 7.4 at 4 °C). Finally, the membrane samples were homogenized using a 23-gauge needle, snap-frozen with liquid nitrogen and stored until further experimentation at −80 °C.

### 2.6 NanoBRET ligand binding assay

NanoBRET binding was measured in triplicate on white low-volume 384-well plates and started by combining 0.3 nM fluorescently labeled CXCL12 (CXCL12-AF647), increasing concentrations of VUF16840 or VUF15485 (10^−5^ M – 10^−11^ M) with 35 ng membranes (protein content) per well of HEK293T cells expressing NLuc-ACKR3. All dilutions were prepared in HBSS supplemented with 0.2% BSA. After assembling the binding reaction, the plate was flash-centrifuged at 100 *g*. Binding reactions were then incubated for 1 hour after which furimazine was added (310 times diluted from stock concentration) to a final volume of 13.5 *μ*L. Total light intensity was measured at 460 nm wavelength with 80 nm bandwidth and separately for wavelengths ≥610 nm using the PHERAstar-FSX (BMG Labtech) with a dual emission filter. The ratio of light intensities (>610 nm over 460 nm) is a measure for the relative binding of CXCL12-AF647 to the NLuc-ACKR3.

### 2.7 [^3^H]VUF15485 radioligand binding assay

Radioligand binding studies were performed as described earlier.^23^ Membranes expressing HA-ACKR3 were incubated with 4 *μ*g membrane protein/well and 2 nM [^3^H]VUF15485 and increasing concentrations VUF16840 or VUF15485, diluted in in HBSS supplemented with 0.2% BSA. All assays were performed in triplicate in a 96-well plate. Binding reactions were incubated for 2 hours at room temperature and then terminated by filtration over a PEI-coated GF/C filter using a cell harvester (Perkin Elmer) followed by 3 consecutive wash steps using ice-cold buffer (50 mM HEPES, 1.2 mM CaCl_2_, 5 mM MgCl_2_, 0.5 M NaCl, pH 7.4 at 4 °C). Filter-bound [^3^H]VUF15485 was measured by adding 25 *μ*L MicroScint-O per well to the dried GF/C-plate and radioactivity was quantified using the Wallac Microbeta counter (Perkin Elmer).

### 2.8 Detection of ACKR3 membrane localization

Ligand-induced receptor mobilization to the plasma membrane was monitored by bystander NanoBRET according to an established method.^36^ For this assay, 5 × 10^6^ HEK293T cells were seeded in 10-cm dishes and cotransfected with plasmids encoding ACKR3 C-terminally tagged with NLuc and mNeonGreen N-terminally tagged with the plasma membrane targeting sequence corresponding to the first 11 residues (MGCIKSKGKDS) of the human Lyn-kinase.^37^ Twenty-four hours after transfection, cells were distributed into black 96-well plates (1 × 10^5^ cells per well) and treated with chemokines (100 nM). After 45-minute incubation at 37 °C, coelenterazine H (10 *μ*M) was added and donor emission (460 nm) and acceptor emission (535 nm) were immediately measured on a GloMax Discover (Promega) plate reader.

Similarly, to determine the concentration-dependent effects of ligand-induced ACKR3 mobilization to the cell membrane, cells were transfected with membrane-targeted mVenus (mVenus-CAAX) and ACKR3-NL (vide supra). Two days after transfection, medium was aspirated and cells were reconstituted in HBSS with 0.05% BSA, 5 *μ*M coelenterazine-h and various concentrations of CXCL11, CXCL12, VUF15485, and/or VUF16840 in triplicate. Cells were subsequently incubated at 37 °C for 20 minutes after which the total light intensity was measured at 475 and 535 nm (both with a bandwidth of 30 nm) using the PHERAstar-FSX with a dual emission filter. The ratio of light intensities (535 nm over 475 nm) is a measure for the relative abundance of ACKR3 residing on the cell membrane.

### 2.9 NanoBiT-complementation assay to measure *β*-arrestin2-ACKR3 and *β*-arrestin2-clathrinA interactions

Two days after transfection (vide supra), medium was aspirated and cells were reconstituted in HBSS with 0.05% BSA. To determine the concentration-dependent effects of VUF16840, VUF15485, and CXCL12, cells were incubated with increasing concentrations ligand at 37 °C for 15 minutes. Similarly, for the inhibition of VUF15485 by VUF16840, 100 nM of VUF15485 was coincubated with increasing concentrations VUF16840 at 37 °C for 15 minutes. After treating the cell with ligand furimazine was added (310 times diluted from stock concentration). Cells were incubating at 37 °C for another 3 minutes before detection of bioluminescent intensities.

To determine the insurmountable antagonism of VUF16840, medium of transfected cells was aspirated and cells were reconstituted in HBSS with 20 mM HEPES and 0.1% BSA. Cells were incubated with VUF16840 or buffer for 60 minutes after which furimazine was added and cells were incubated for another 5 minutes, all at 37 °C. Subsequently increasing concentrations of CXCL11 or CXCL12 were added and bioluminescent intensities were measured at 25 °C. The bioluminescent intensities after 30 minutes are depicted.

Bioluminescence was measured using the PHERAstar-FSX and is a measure for the relative proximity of LgBiT- and SmBiT-tagged proteins and thus the occurrence of investigated protein–protein interaction.^38^

### 2.10 NanoBiT-complementation based chemokine receptor screen

The pharmacological modulation of all chemokine receptors by VUF16840 was performed according to an established method.^8^^,^^23^^,^^39^ Briefly, *β*-arrestin2 recruitment to chemokine receptors in response to 1 *μ*M VUF16840 or positive control chemokines was monitored by NanoLuc complementation assay (NanoBiT, Promega). Four hours after transfection, cells were harvested, incubated for 25 minutes at 37 °C with furimazine and distributed into a white 96-well plates (1 × 10^5^ cells per well). *β*-Arrestin2 recruitment was then detected for 20 minutes upon treatment with 1 *μ*M VUF16840 (or increasing concentrations ligand for experiments with CC chemokine receptor [CCR3]) using the Mithras LB940 luminometer (Berthold Technologies). To evaluate the antagonist properties of VUF16840, full agonists of each receptor were added after the 20-minute incubation with VUF16840. Signal from wells treated with full agonist only was defined as 100% activity and signals from wells treated with no agonist were used to set 0% activity.

### 2.11 CXCL12 uptake in ACKR3-overexpressing hCMEC/D3 cells

Human CMEC (D3) cells (CVCL_U985) stably overexpressing ACKR3 and empty vector control were generated by retroviral transductions. The lentiviral expression vector encoding the human ACKR3 was created by cloning the ACKR3 gene (NM_020311.3 from GenScript) in the restriction sites Nhe1/Sma1 of the pLV-LifeAct (a kind gift of Dr Niels Heemskerk). Lentiviral particles were packaged in HEK293T cells, cultured in Dulbecco’s modified Eagle’s medium containing 10% fetal calf serum, 1% penicillin/streptomycin (ThermoFisher Scientific) at 37 °C in a 5% CO_2_ incubator. Lentiviral expression and packaging vectors were introduced using calcium phosphate transfection. Supernatant containing virus was collected and virus was concentrated by centrifugation (Hettich swing-out rotor, 250 × *g*), aliquoted (Amicon Ultra15, UFC910024 Merck) and stored at −80 °C. For transduction of hCMEC/D3 endothelial cells, virus-containing supernatant was added dropwise to hCMEC/D3 cells in a 24 well-microplate. Virus supernatant was replaced with appropriate medium after 24 hours incubation. Transduced cells were selected using puromycin (1:2000). For uptake assays, D3-ACKR3+ cells were grown to confluence in 96 wells plates in EG-2MV medium (Lonza; 1#CC3202). Cells were pretreated for 30 minutes with 1, 10, or 100 nM VUF16840, where after 3 nM CXCL12-A647 (Protein Foundry) was added to the medium for 60 minutes. Supernatant was taken off, cells were washed 1× with medium and CXCL12-A647 signal in cells was measured using a BioTek Cytation microplate reader (Agilent). For immunocytochemistry, D3-ACKR3+ cells were cultured and stimulated as above on 8 well *μ*-slides (Ibidi Systems), where after they were fixed with 4% paraformaldehyde for 10 minutes. Cells were washed with PBS and permeabilized for 5 minutes in PBS + 0.05% Triton-X after they were blocked with 10% goat serum for 60 minutes at room temperature. Rhodamine phalloidin (Molecular Probes, #R415; 1:400) was added for 1 hour, after which cells were washed, incubated with Hoechst (1:1000) for 5 minutes, washed and covered with mowiol on coverslips. Images were taken using a confocal microscope at 40× magnification.

### 2.12 Data analysis

The sigmoidal concentration-dependent displacement of binding of the probes [^3^H]VUF15485 and CXCL12-A647 by unlabeled ligands were analyzed using GraphPad Prism 8, by fitting a one-site binding model to the data, to determine the pIC_50_. Mean pIC_50_ values were determined by averaging fitted values from individual experiments. Graphs depict the pooled data of all experiments. Similarly, sigmoidal, ligand-induced concentration-dependent activation or inhibition of the proximity between ACKR3 and the protein of interest was analyzed using GraphPad Prism 8, by fitting a 3-parameter concentration-response model to the data, to determine pEC_50_/pIC_50_ values. Mean pEC_50_ values and pIC_50_ values were determined by averaging fitted values from individual experiments. Graphs depict the pooled data of all experiments, for which values are normalized, per experiment, to the fold difference compared with basal signal. CXCL12 uptake and uptake inhibition by endothelial cells was analyzed with GraphPad Prism 10.2.0 using one-way analysis of variance.

## 3 Results

### 3.1 VUF16840 binds ACKR3 with nanomolar affinity

The ACKR3 ligand VUF16840 was evaluated for its binding to hACKR3 in different competition binding assay formats (Fig. 1). Binding of fluorescent Alexa Fluor 647-labeled CXCL12 (CXCL12-AF647) to membranes expressing N-terminally NLuc-tagged hACKR3 was quantified by a homogeneous NanoBRET binding assay,^40^ whereas binding of [^3^H]VUF15485 to HA-tagged hACKR3 expressing membranes was determined after filtration of incubation mixtures and scintillation counting.^23^ Coincubation of CXCL12-A647 and increasing concentrations of VUF16840 or the high-affinity small-molecule ACKR3 agonist VUF15485^23^ resulted in a concentration-dependent decrease in CXCL12-A647 binding to membranes expressing the NLuc-hACKR3 (Fig. 1A). Both unlabeled ligands displaced CXCL12-A647 binding to NLuc-hACKR3 to similar levels with high inhibitory potencies; VUF15485 displaced CXCL12-A647 binding to NLuc-hACKR3 with a pIC_50_ value of 7.6 ± 0.1 (mean ± SD, *n* = 3; IC_50_ = 28 nM), whereas VUF16840 showed a pIC_50_ value of 8.2 ± 0.1 (mean ± SD, *n* = 3; IC_50_ = 7 nM). Both compounds were also potent in displacing the binding of [^3^H]VUF15485 from HA-hACKR3 expressing membranes. As shown in Fig. 1B, VUF16840 fully displaced [^3^H]VUF15485 binding from hACKR3 with a pIC_50_ of 9.1 ± 0.0 (mean ± SD, *n* = 13; IC_50_ = 0.7 nM). As previously shown,^23^ VUF15485 potently displaced [^3^H]VUF15485 binding from the hACKR3 with a pIC_50_ of 7.7 ± 0.1 (mean ± SD, *n* = 18; IC_50_ = 20 nM).

### 3.2 VUF16840 inhibits constitutive *β*-arrestin2 recruitment to ACKR3

**Fig. 2:**
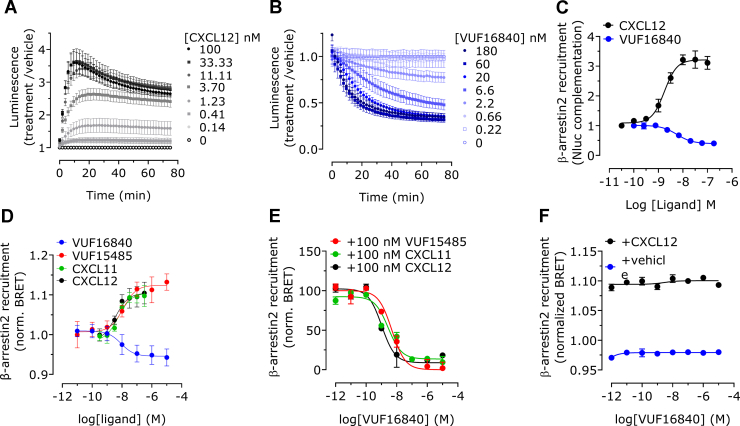
VUF16840 inhibits constitutive as well as agonist induced *β*-arrestin2 recruitment to ACKR3. (A) Transiently transfected HEK293T cells, expressing hACKR3-SmBiT and *β*-arrestin2-LgBiT, were stimulated with increasing concentrations CXCL12 or (B) VUF16840. Subsequently, *β*-arrestin2 recruitment to hACKR3 was detected by NanoBiT complementation. (C) Transfected HEK293T cells were stimulated for 30 minutes with increasing concentrations CXCL12 or VUF16840 and *β*-arrestin2 recruitment to the hACKR3 was detected by NanoBiT complementation. (D) Transfected HEK293T cells were stimulated with increasing concentrations VUF15485, VUF16840, CXCL11 or CXCL12, and *β*-arrestin2 recruitment to hACKR3 was detected by BRET. (E) *β*-Arrestin2 recruitment to the ACKR3 was measured after stimulation with 100 nM of VUF15485, CXCL11 or CXCL12 in the presence of increasing concentrations VUF16840. (F) Transfected HEK293T cells were stimulated with increasing concentrations VUF16840 in the presence or absence of 100 nM CXCL12 and *β*-arrestin2 recruitment to the hCXCR4 was detected by biolumescence resonance energy transfer. Depicted data is normalized as fold-basal and represents the mean ± SD of n experiments with triplicate measurements per experiment. Panel A–C and F depicts the average of 3 experiments; for panel D and E, the number of experiments differed per condition and is summarized in Supplemental Table 1.

In the absence of G protein coupling, ACKR3 activation upon ligand binding is typically assessed by monitoring *β*-arrestin2 recruitment.^36^ VUF16840 was therefore tested in a NanoBiT-based hACKR3-*β*-arrestin2 recruitment assay (Fig. 2). As expected, addition of the ACKR3 agonist CXCL12 (0.14–100 nM) to HEK293T cells transiently expressing hACKR3-SmBiT and *β*-arrestin2-LgBiT resulted in a rapid and concentration-dependent bioluminescence signal, indicative of successful NLuc complementation. Although low concentrations of CXCL12 produced a stable signal, higher agonist concentrations lead to a signal that gradually reduced over time (Fig. 2A), possibly due to increased ACKR3 internalization (vide infra).

In the same experimental set-up, VUF16840 did not lead to an increase in hACKR3 *β*-arrestin2 recruitment, but interestingly, the ligand time and concentration-dependently inhibited *β*-arrestin2 recruitment, suggesting constitutive coupling of hACKR3 to *β*-arrestin2 (Fig. 2B). Analysis of concentration-response curves generated after 30 minutes of incubation resulted in a pEC_50_ value of 8.8 ± 0.2 (mean ± SD, *n* = 3; EC_50_ = 1.6 nM) for the endogenous agonist CXCL12 and a pIC_50_ value of 8.2 ± 0.1 (mean ± SD, *n* = 3; IC_50_ = 6.3 nM) for the inverse agonist VUF16840 (Fig. 2C).

In an orthogonal BRET-based assay, HEK293T cells were transiently transfected with *β*-arrestin2-mVenus and hACKR3-Rluc8, allowing detection of *β*-arrestin2 recruitment to hACKR3 by BRET. As observed before,^23^ the endogenous agonists CXCL11 and CXCL12 and the synthetic small molecule ACKR3 agonist VUF15485 induced concentration-dependent recruitment of *β*-arrestin2 to hACKR3 with nanomolar potencies (Fig. 2D; Supplemental Table 1). In contrast, VUF16840 induced a concentration-dependent reduction of *β*-arrestin2 recruitment to hACKR3 with a pIC_50_ value of 8.0 ± 0.1 (mean ± SD, *n* = 7; IC_50_ = 10 nM). The data obtained in this orthogonal assay corroborate the earlier findings of VUF16840 acting as an inverse agonist and hACKR3 showing constitutive coupling to *β*-arrestin2. The observed inverse agonism in a *β*-arrestin2 recruitment assay has so far not been reported for ACKR3 ligands and constitutive recruitment of a *β*-arrestin2 has so far only been incidentally reported for other GPCRs.^41^, ^42^, ^43^, ^44^

**Fig. 3:**
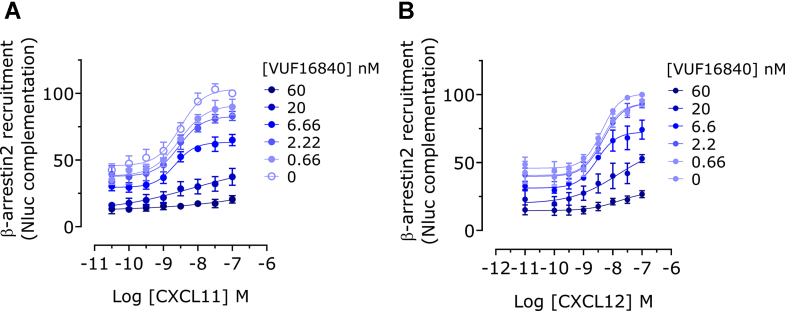
VUF16840 noncompetitively inhibits agonist-induced *β*-arrestin2 recruitment to hACKR3. (A) Transiently transfected HEK293T cells, expressing hACKR3-SmBiT and *β*-arrestin2-LgBiT were stimulated with increasing concentrations of (A) CXCL11 or (B) CXCL12 following a 60 minutes pretreatment with buffer or various concentrations of the inverse agonist VUF16840. Next, the *β*-arrestin2 recruitment to the hACKR3 was detected by NLuc complementation. Depicted data are the mean ± SD of 3 experiments.

In line with the aforementioned binding experiments with labeled CXCL12, VUF16840 competed with ACKR3 agonists in a *β*-arrestin2 recruitment assay (Fig. 2E). The inverse agonist exhibited a full concentration-dependent inhibition of ACKR3-*β*-arrestin2 interactions, induced by both CXCL11 and CXCL12 chemokines, as well as the small molecule agonist VUF15485 (100 nM) (Fig. 2E; Supplemental Table 1). However, VUF16840 did not affect *β*-arrestin2 recruitment to the related CXCR4 receptor, neither in the presence nor absence of the shared, endogenous agonist CXCL12 (Fig. 2F). In a more detailed analysis (Fig. 3), ACKR3 expressing cells were pretreated with different concentrations of VUF16840 and concentration-response-curves of the ACKR3 ligands CXCL12 and CXCL11 were measured (Schild analysis). In this assay set-up, VUF16840 inhibited both CXCL12- and CXCL11-mediated *β*-arrestin2 recruitment in a nonsurmountable fashion (Fig. 3). Whereas there is no actual shift in the EC_50_ values for the chemokines observed, the maximal response at high agonist concentrations is reduced concentration-dependently.

### 3.3 Chemokine receptor selectivity of VUF16840

**Fig. 4:**
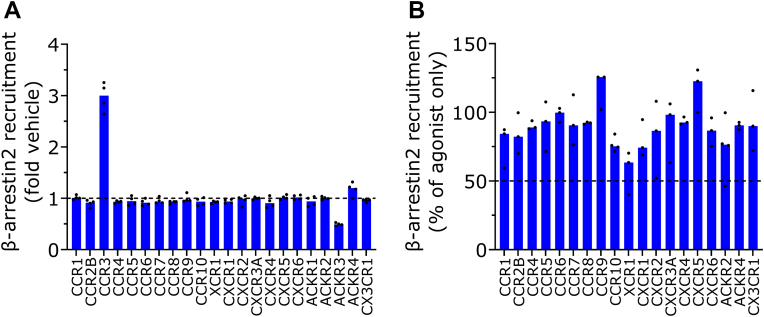
Modulation of a panel of chemokine receptors by VUF16840. *β*-Arrestin2 recruitment toward a full panel of 19 chemokine receptor was measured using a NLuc complementation assay in HEK293T cells. In panel A, VUF16840 (1 *μ*M) mediated effect on *β*-arrestin2 recruitment is depicted, whereas in panel B, VUF16840 (1 *μ*M) was coincubated with a receptor specific chemokine. Values depict the mean ± SD of 4 experiments for CCR10 and ACKR2 and 3 experiments for all other receptors in panel A. Panel B depict the mean ± SD of 4 experiments.

To verify whether inverse agonism by VUF16840 is hACKR3 specific, the ligand was investigated for agonistic and antagonistic properties toward a panel of 19 human chemokine receptors (Fig. 4). To achieve this, HEK293T cells were transiently transfected with each chemokine receptor, C terminally tagged with the SmBiT NanoBiT sensor in combination with *β*-arrestin2, N-terminally fused to the LgBiT sensor. Transfected cells were treated with a high concentration (1 *μ*M) VUF16840 (agonist mode screen, Fig. 4A) or with a combination of the respective agonist of each receptor together with VUF16840 (antagonist-mode screen, Fig. 4B). In the agonist assay set-up VUF16840 stimulation only modulated *β*-arrestin2 recruitment to hACKR3 and hCCR3 (Fig. 4A). As expected, VUF16840 inhibited basal *β*-arrestin2 recruitment to hACKR3. Surprisingly, VUF16840 acted as an agonist for CCR3-mediated *β*-arrestin2 recruitment (Fig. 4A). Full concentration-response analysis (Supplemental Fig. 5) showed that VUF16840 was able to activate CCR3 with submicromolar potency (pEC_50_ = 6.7 ± 0.1; EC_50_ = 200 nM), whereas its endogenous ligand CCL13 activated CCR3 in our hands with almost equal potency (pEC_50_ = 7.2 ± 0.1; EC_50_ = 63 nM). Interestingly, VUF16840 activates CCR3 with higher efficacy (1.4-fold) than the endogenous agonist CCL13. In an antagonist-mode assay, VUF16840 was coincubated with the respective endogenous agonist and in this set-up it did not show extensive inhibition (>50%) of any of the tested chemokine receptors (Fig. 4B). These data confirm that VUF16840 is at least 30-fold specific over CCR3 and shows an even larger selectivity over other tested chemokine receptors. It is anticipated that VUF16840 only modulates ACKR3 and CCR3 at pharmacologically relevant concentrations.

### 3.4 VUF16840 inhibits constitutive ACKR3 signaling and trafficking events

**Fig. 5:**
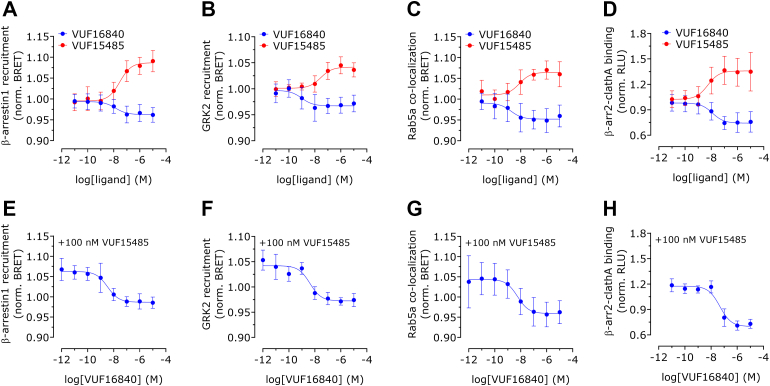
Inhibition of both constitutive as well as agonist-induced ACKR3 activity by VUF16840. HEK293T cells were stimulated with increasing concentrations of VUF15485 or VUF16840 only (A–D), or increasing concentrations of VUF16840 in the presence of 100 nM VUF15485 (E–H). The resulting proximity between ACKR3 and *β*-arrestin1 (A, E), GRK2 (B, F), or Rab5a (C, G) was determined by bioluminescence resonance energy transfer. Furthermore, an interaction between clathrin A and *β*-arrestin2 was measured by NLuc complementation, as was determined by quantifying the relative light units (D, H). Depicted data is normalized as fold-basal and represents the mean ± SD of N experiments with triplicate measurements per experiment. N is specified in Supplemental Table 1.

Next, it was explored whether VUF16840 could also affect a number of additional ACKR3 signaling or trafficking events. Both *β*-arrestin1 and GRK2 are known to be recruited to hACKR3 upon activation,^10^^,^^23^^,^^45^,^46^ and phosphorylation of the C-terminus is found to be crucial for ACKR3 internalization and *β*-arrestin1/2 recruitment,^10^^,^^47^^,^^48^ mediated at least in part by GRK2.^10^^,^^47^ Additionally, clathrin A is a structural protein involved in the formation of clathrin-coated vesicles mediating receptor packaging and internalization, whereas Rab5a is a protein highly expressed in early endosomes.^32^^,^^49^ In order to explore how VUF16840 affects some of the events related to ACKR3 trafficking, BRET sensors in transiently transfected HEK293T cells were used to explore ACKR3 interaction with the following intracellular proteins: *β*-arrestin1, *β*-arrestin2, GRK2, Rab5a, and clathrin A (Fig. 5). The ACKR3 agonist VUF15485 induced recruitment of *β*-arrestin1 and GRK2 (Fig. 5, A and B) as well as translocation of ACKR3 toward early endosomes (Fig. 5C), all with similar, high potencies (pEC_50_ values of 7.4–8.1; Supplemental Table 1). Agonist-induced internalization of ACKR3 was further monitored by measuring the interaction between clathrin A and *β*-arrestin 2 using a NanoBiT-based protein–protein interaction assay (Fig. 5D). This interaction marks clathrin A-mediated endocytosis of GPCR bound *β*-arrestin2^32^ and was stimulated in a concentration-dependent manner by VUF15485 (pEC_50_ = 8.2 ± 0.4, mean ± SD, *n* = 5; EC_50_ = 6 nM). In all these assay setups VUF16840 acted as an inverse agonist and inhibited the constitutive ACKR3-mediated responses with nanomolar potencies (pEC_50_ values of 8.0–8.8; Supplemental Table 1). In line with the effect of VUF16840 on *β*-arrestin2 recruitment to the ACKR3, VUF16840 could also fully inhibit (Figs. 5, E–H) VUF15485-responses (pIC_50_ values 7.3–8.7; Supplemental Table 1).

**Fig. 6:**
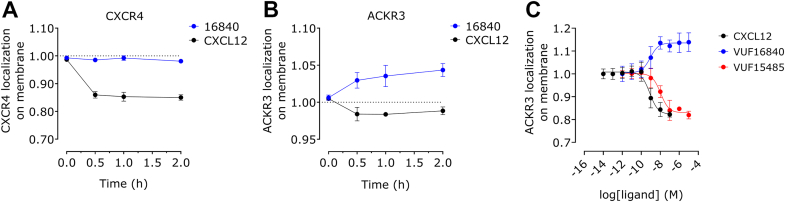
CXCL12 and VUF16840 differentially affect cell surface localization of hACKR3-NLuc. (A) Transiently transfected HEK293T cells, coexpressing hACKR3-NLuc and membrane-targeted mNeonGreen were incubated with 100 nM CXCL12 or 1 *μ*M VUF16840. The resulting proximity between hACKR3-NLuc and was determined as bystander BRET. Data depict the mean ± SD of 4 (VUF16840) and 2 (CXCL12) experiments. (B) The same assay was performed as in A for hCXCR4-NLuc. Data depict the mean ± SD of 4 (VUF16840) and 2 (CXCL12) experiments. (C) Transiently transfected HEK293T cells, coexpressing hACKR3-NLuc and mVenus-CAAX were incubated for 15 minutes with increasing concentrations CXCL12 or VUF16840. Expression of hACKR3-NLuc at the cell surface was determined by the measurement of bystander BRET. Depicted data represents the mean ± SD of 3 experiments with triplicate measurements per experiment.

ACKR3 cell surface expression was also studied by a bystander BRET method^37^ in which plasma membrane colocalization of hACK3-NLuc and membrane-targeted mVenus or mNeonGreen was measured (Fig. 6). As shown in Fig. 6A, CXCL12 exposure of hACKR3-NLuc expressing HEK293T cells resulted in a rapid reduction of bystander BRET signal from plasma membrane-targeted mNeonGreen, indicative hACKR3-NLuc internalization. Exposure of these cells to the inverse agonist VUF16840 increased the bystander BRET signals, indicative of receptor accumulation on the cell surface. In a similar set-up with hCXCR4-NLuc, CXCL12 exposure also resulted in (sustained) CXCR4 internalization, but CXCR4 surface expression was not affected by VUF16840 (Fig. 6B). Moreover, bystander BRET between ACKR3-NLuc and plasma membrane localized mVenus-CAAX was concentration-dependently inhibited by CXCL12 (pIC_50_ = 9.1 ± 0.2, mean ± SD, *n* = 3) and increased by VUF16840 (pIC_50_ = 9.1 ± 0.2, mean ± SD, *n* = 3).

### 3.5 The inverse agonist VUF16840 blocks CXCL12 uptake in ACKR3 expressing endothelial cells

**Fig. 7:**
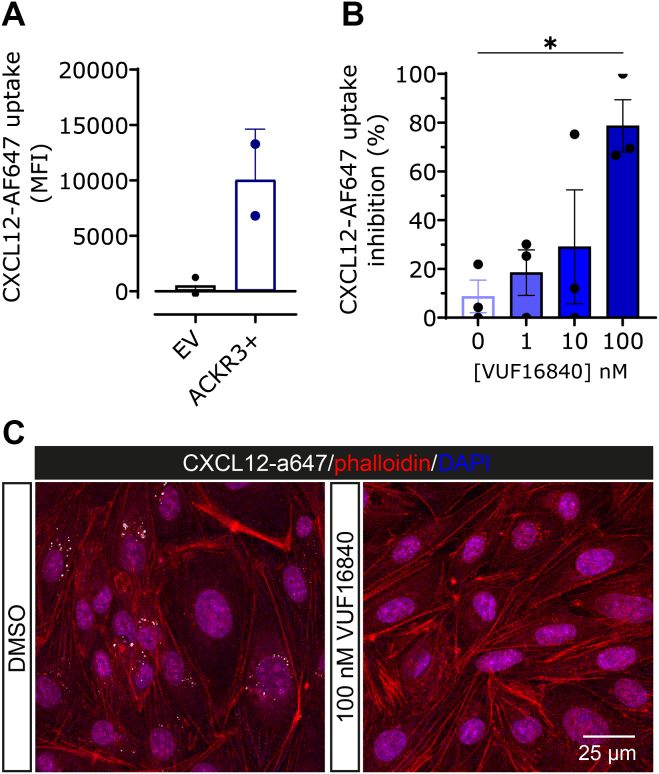
Fluorescent CXCL12-AF647 uptake by hACKR3-expressing D3 endothelial cells. (A) Stably transduced D3-ACKR3+ cells were incubated with 3 nM CXCL12-AF647 and fluorescent chemokine uptake in control cells [transfected with empty vector (EV)] and ACKR3+ cells was measured after 60 minutes as the mean fluorescent intensity (MFI). Data depict the mean ± SD of 2 experiments. (B) Uptake of CXCL12-AF647 in D3-ACKR3+ cells in the absence or presence of increasing concentrations VUF16840. Depicted data represents the mean ± SD of 3 experiments. Comparisons marked with ∗ indicates a significant difference of *P* < .05 according to an ordinary one-way analysis of variance. (C) D3-ACKR3+ cells incubated with 3 nM CXCL12-AF647 in the absence (DMSO) or presence of 100 nM VUF16480, were fixed and stained with phalloidin (red, actin filaments) and 4′,6-diamidino-2-phenylindole (DAPI, blue, nuclei) and imaged by confocal microscopy for CXCL12-AF647 uptake (white dots).

Because VUF16840 inhibits CXCL12 binding to hACKR3 and affects also its constitutive internalization dynamics, we hypothesized that VUF16840 would also affect chemokine uptake via hACKR3. To evaluate the uptake of CXCL12 via ACKR3 internalization, we stably expressed hACKR3 in the endothelial cell line Human CMEC (D3) via retroviral transduction. As shown in Fig. 7A, the resulting D3-ACKR3+ cell line indeed internalized CXCL12-A647. After 60 minutes. incubation of D3-ACKR3+ cells with 3 nM CXCL12-AF647, significant intracellular accumulation of CXCL12-AF647 was observed. In line with the observed inhibition of ACKR3 internalization, coincubation with VUF16840 resulted in concentration-dependent inhibition of CXLC12-AF647 uptake (Fig. 7B). After incubation of D3-ACKR3+ cells with 3 nM CXCL12-AF647 in the absence (DMSO) or presence of 100 nM VUF16840, cells were fixed and stained for actin and DNA with rhodamine-labeled phalloidin (red) and 4′,6-diamidino-2-phenylindole (DAPI; blue), respectively (Fig. 7C). In the absence of VUF16840 CXCL12-A647 uptake could be observed (white dots), whereas VUF16840 treatment (100 nM) abolished the uptake of CXCL12-AF647 (Fig. 7C).

## 4 Discussion

In this study we describe the pharmacological properties of a new high-affinity ACKR3 ligand, VUF16840. This compound combines nanomolar binding affinity for ACKR3 (Fig 1B; Supplemental Table 1) with nonsurmountable inhibition of chemokine-induced receptor activation, measured as ACKR3-*β*-arrestin2 interactions (Fig. 3; Supplemental Table 1). At high concentrations, VUF16840 completely prevents both CXCL11 and CXCL12 activation of ACKR3. A similar effect of VUF16840 is observed for ACKR3 inhibition after activation with the small molecule agonist VUF15485 (Supplemental Fig. 6). Previously, most ACKR3 modulators with translational potential (eg, CCX777 or CCX733) have been reported as agonists in ACKR3-dependent *β*-arrestin2 recruitment assays^21^^,^^24^ and only recently ACKR3 antagonists were reported.^50^ The chemical series of VUF16840 was originally identified within patent literature^25^ and during the course of this study compounds from the patent were published as nonsurmountable ACKR3 antagonists.^26^^,^^27^ Moreover, the selected clinical candidate from this series, ACT1004-1239, was reported to have therapeutic potential in animal models of MS and has entered clinical trials.^28^, ^29^, ^30^ Additionally, a different series of moderately active ACKR3 antagonists for the recruitment of *β*-arrestin2 was also reported by Pfizer recently.^50^ However, neither these ligands, nor ACT1004-1239 were shown to stabilize an inactive conformation of ACKR3, as is clearly the case for VUF16840 in our hands (Figs. 2, A and C and 4, A–D). These data are corroborated by a recent manuscript reporting on single molecule analysis of the conformational landscape of ACKR3 using VUF16840 as tool.^51^ In the present study VUF16840 not only blocks chemokine-induced ACKR3 activation, but also inhibits constitutive recruitment of GRK2, *β*-arrestin1 and *β*-arrestin2 to ACKR3, next to constitutive interaction of ACKR3 with Rab5a and *β*-arrestin2 with clathrin A. The inverse agonism displayed by VUF16840 also affects ACKR3 expression at the plasma membrane (Fig. 6). As reported previously,^52^ ACKR3 traffics constitutively from the plasma membrane to Rab5a-expressing early endosomes (Fig. 5C), most likely via clathrin A-coated pits (Fig. 5D). Stimulation with agonists, such as CXCL12 can further increase the level of ACKR3 internalization, whereas exposure to the inverse agonist VUF16840 leads to its enhanced cell surface expression as measured by bystander BRET.

Inverse agonism by direct VUF16840 analogs, such as ACT1004-1239, has so far not been reported.^26^ This is not necessarily the consequence of structural and related pharmacological differences between those compounds and VUF16840, but potentially reflects differences in assay methodology, as the detection of constitutive GPCR signaling is known to rely on factors such as cellular background and GPCR expression levels.^53^^,^^54^ Constitutive activity, the ability of GPCRs to signal in the absence of an external ligand, has emerged as a key feature influencing receptor physiology and pharmacology.^53^^,^^54^ This phenomenon reflects the intrinsic capability of GPCRs to adopt active conformations spontaneously, enabling basal levels of signaling. Constitutive activity has been documented across various GPCR families, including (mutant) human chemokine receptors, such as CCR1,^54^ CCR5,^55^ CXCR3,^56^ or CXCR4.^57^ Consequently, several drug molecules targeting these receptors have been shown to act as inverse agonists.^54^, ^55^, ^56^, ^57^ A number of viral chemokine GPCRs (eg, ORF74 and US28) have previously also been shown to display a very high level of constitutive activity.^58^ For both CCR1 and the viral GPCR US28 the high level of constitutive signaling has been linked to increased levels of intracellular expression of the GPCR protein and internalization via *β*-arrestin-mediated mechanisms,^54^^,^^58^ as in the case of ACKR3.

The constitutive recycling of ACKR3, similarly to the recycling of viral receptor US28,^58^^,^^59^ has been linked to cellular uptake of chemokine ligands.^49^ ACKR3 has therefore been hypothesized to act as a “chemokine sink” and indirectly affecting CXCR4 signaling.^9^^,^^10^^,^^47^^,^^49^^,^^60^ In line with VUF16840 inhibiting constitutive recycling of ACKR3, the inverse agonist also completely blocks the in vitro uptake of fluorescent CXCL12 in endothelial cells stably overexpressing ACKR3. In addition, in vivo application of ACT1004-1239 has been shown to increase plasma and brain CXCL12 levels in mice^28^^,^^30^ and plasma levels in humans,^61^ highlighting a potential relevant therapeutic effect of blocking the constitutive internalization of ACKR3.

In a selectivity screen with a wide panel of chemokine receptors, VUF16840 is shown to selectively interact with ACKR3 over most other chemokine receptors (Fig. 4). The only observed off-target effect for VUF16840 was moderate agonistic activity on the CCR3 (pEC_50_ 6.7). Some features of VUF16840 indeed resemble those of high-affinity CCR3 antagonists, such as the haloarene, piperidine, and amide groups of AZD3778, Banyu(l), GW766994, and lazucirnon.^62^ Despite a large difference in EC_50_ concentrations (>30-fold selectivity), the cross-reactivity of VUF16840 at CCR3 warrants caution in complex in vivo models, including the application of commercially available CCR3 antagonists (eg, SB297006) to offset potential CCR3-activation by the VUF16840.

In a related study we recently combined hydrogen/deuterium exchange mass spectrometry, site-directed mutagenesis, and molecular dynamics simulations to show that VUF16840 and VUF15485 seem to largely occupy similar but distinct binding pockets of ACKR3.^63^ Interestingly, this makes the insurmountable antagonism of VUF15485 by VUF16840 (Supplemental Fig. 6) unexpected, as we would expect VUF16840 to be displaced by high concentrations of VUF15485 if they share the same binding site. Because several cryo-EM structures have in the meantime been resolved for peptide and small-molecule agonist-ACKR3 complexes^64^ or a *β*-arrestin2 x-ray structure bound to the C-tail of ACKR3^65^ structural biology approaches might in the future shed more light on the actual binding pocket of inverse agonists, such as VUF16840. Interestingly, the inverse agonistic properties of VUF16840 on the ACKR3 were further explored in a single molecule Förster resonance energy transfer analysis^51^ showing a clearly distinct conformational landscape of the receptor after binding of VUF16840 compared with VUF15485. VUF16840 was shown to enrich a distinct population of ACKR3 conformations, which, conversely, was destabilized upon binding of VUF15485. To understand the mechanism of inverse agonism and the noncompetitive inhibition of agonist responses at the ACKR3 by VUF16840, it would be of great interest to get a more structural understanding of this inactive receptor state in the future.

In conclusion, our findings highlight VUF16840 as a potent and selective inverse agonist of ACKR3 with the ability to block both constitutive and ligand-induced receptor activation, internalization, and CXCL12 uptake. By stabilizing an inactive conformation, VUF16840 alters ACKR3’s trafficking dynamics, leading to enhanced plasma membrane localization and inhibition of its “chemokine sink” function. The compound’s selectivity for ACKR3, alongside its inverse agonistic properties, underscores its utility as a valuable tool for studying ACKR3 biology and pharmacology. Future research into the structural mechanisms underlying inverse agonism at ACKR3, including the precise binding interactions of VUF16840, will be instrumental in advancing therapeutic strategies targeting this atypical receptor in pathologic conditions.

## Conflict of interest

The authors declare no conflicts of interest.
