# Supplementary material for: Inhibition of constitutive activity of the atypical chemokine receptor 3 by the small-molecule inverse agonist VUF16840
Source: Mol Pharmacol. 2025 Oct 26;107(12):100085. doi: 10.1016/j.molpha.2025.100085 (PMC12799421; doi:10.1016/j.molpha.2025.100085)
Supplement: Supplementary Material [file mmc1.docx]

Supplementary information

**Inhibition of constitutive activity of the atypical chemokine receptor ACKR3 by the small-molecule**

**inverse agonist VUF16840**

Reggie Bosma^1^, Desislava Nesheva^1^, Merel Rijnsburger^2^, Rick Riemens^1^, Justyna M. Adamska^1^, Max Meyrath^3^, Simon Mobach^1^, C. Maurice Buzink^1^, Suzanne van der Pol^2^, Iwan J. P. de Esch^1^, Martyna Szpakowska^3^_,_ Maikel Wijtmans^1^, Andy Chevigne^3^, Henry F. Vischer^1^, Rob Leurs^1*^

**Supplemental Figure 1:** Synthetic scheme for VUF16840. This scheme was based with adaptations on a patent disclosure by Idorsia Pharmaceuticals. Details for the synthesis of a similar compound (clinical candidate ACT-1004-1239) were published during the course of our study^1,2^. ON=overnight. RT=room temperature.


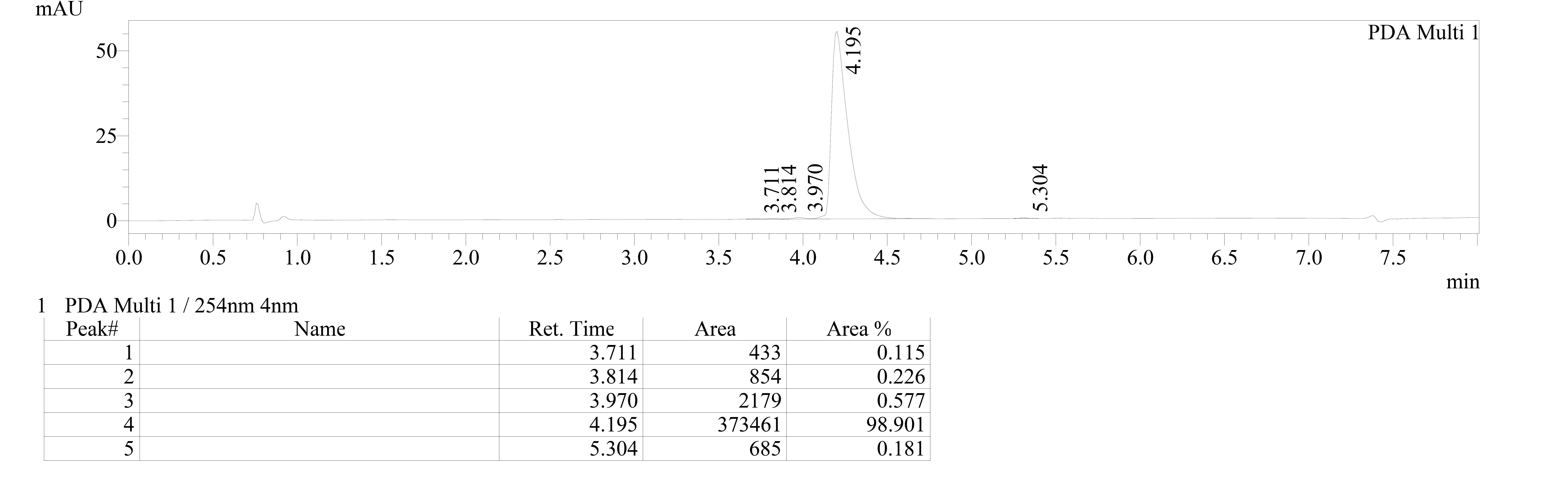


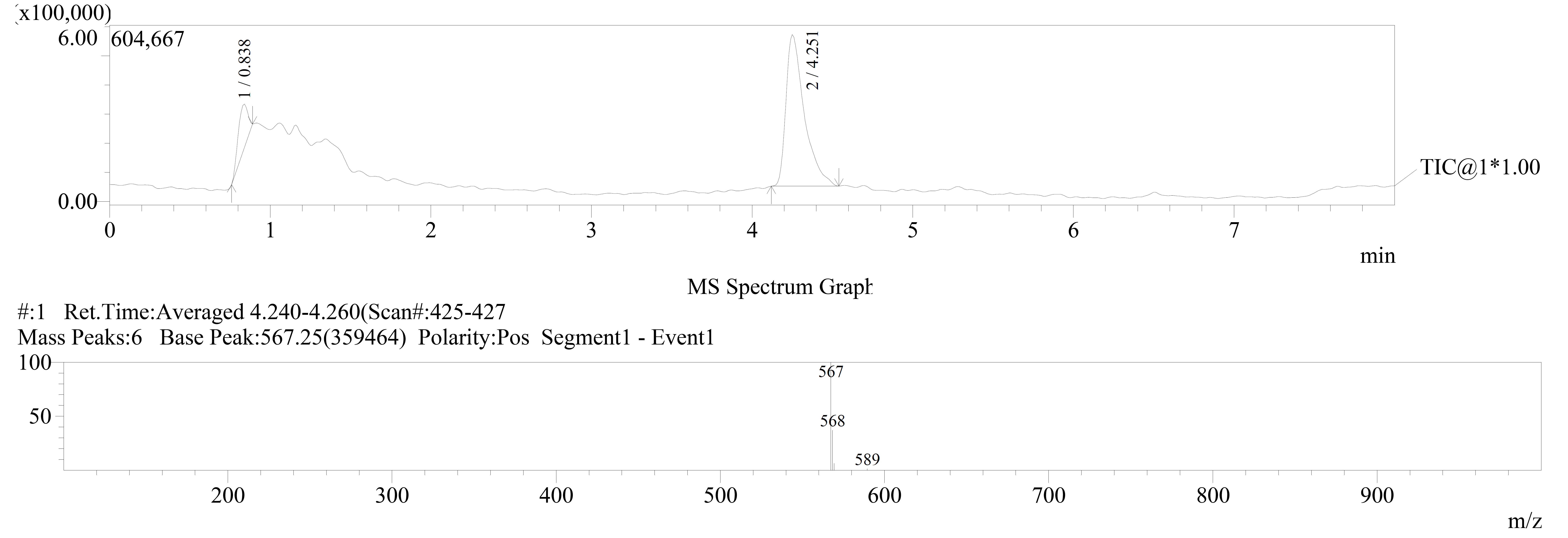


**Supplemental Figure 2**: LC-MS analysis of VUF16840

**
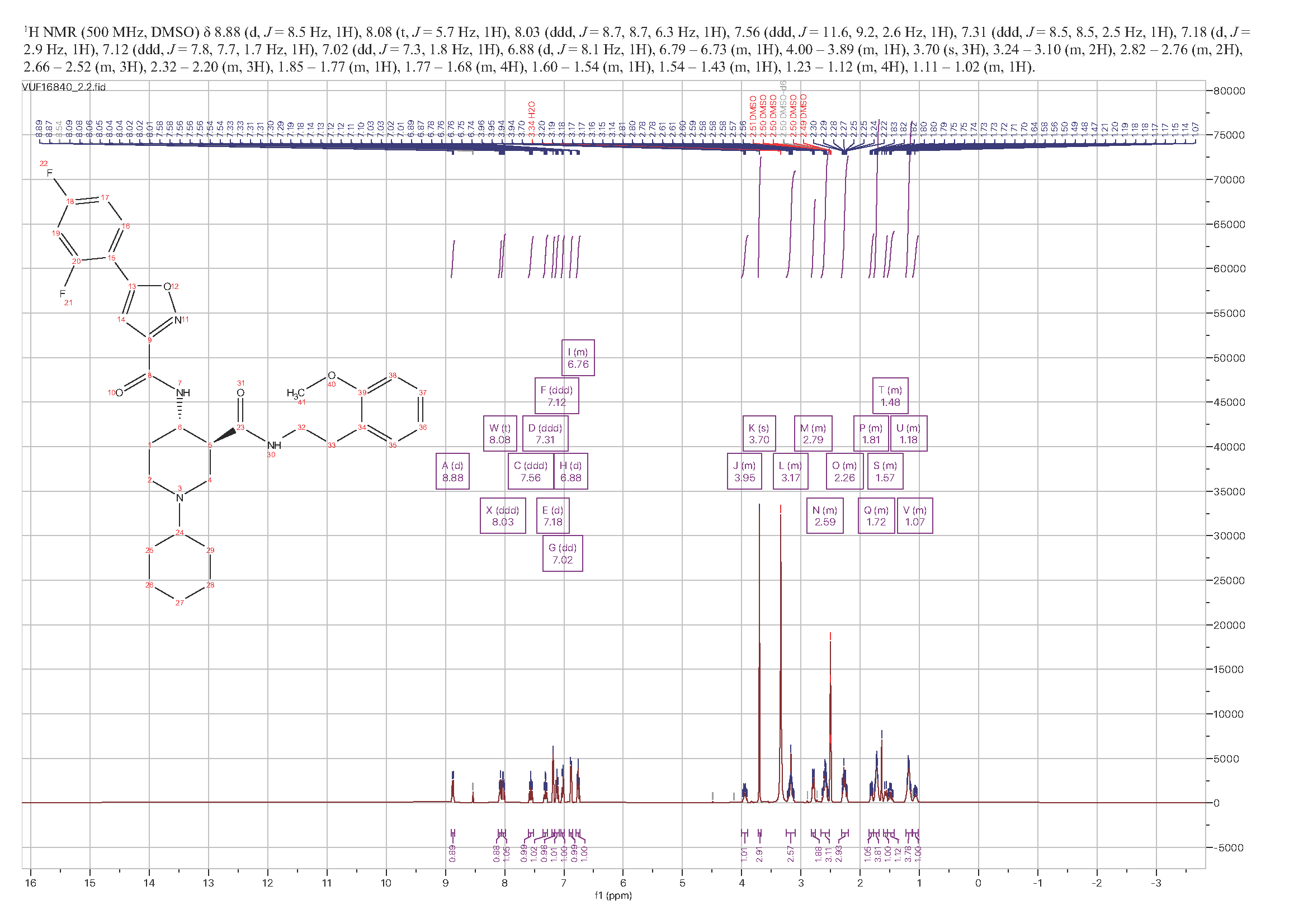
**

**Supplemental Figure 3**: ^1^H NMR spectrum of VUF16840 (DMSO-d6)


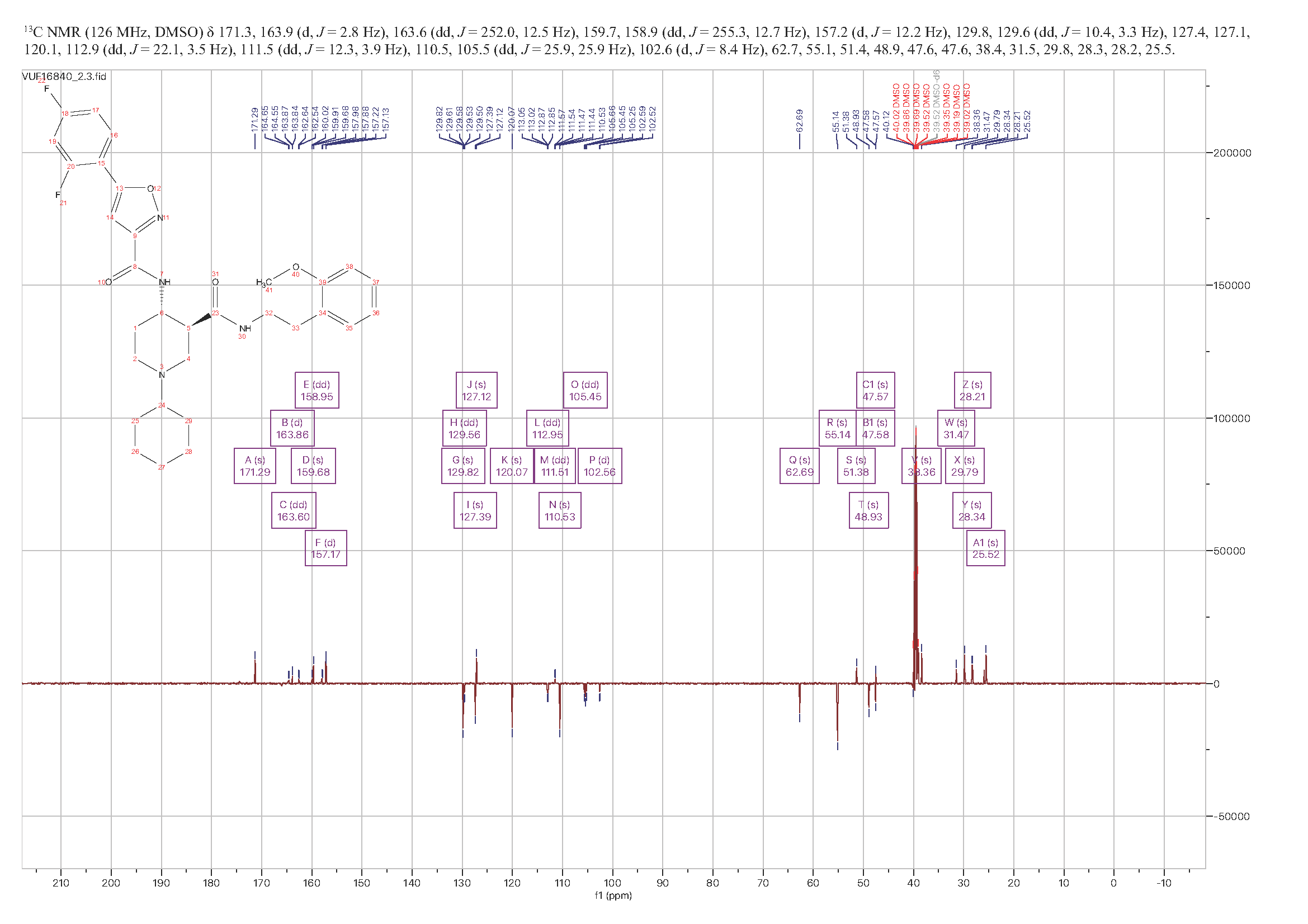


**Supplemental Figure 4**: ^13^C NMR spectrum of VUF16840 (DMSO-d6)

**Supplemental Figure 5 – Modulation of CCR3 receptor by VUF16840.** Recruitment of β-arrestin2 towards CCR3 chemokine receptor was measured using a NLuc complementation assay in HEK293T cells. The concentration-dependent activation of the CCR3 was determined for VUF16840 and the endogenous agonist CCL13. All values depict the mean ± SD of ≥ 3 experiments.

**Supplemental Figure 6 – Modulation of VUF15485-mediated ACKR3 activation by VUF16840.** Transfected HEK293T cells (ACKR3-Rluc8 and β-arrestin2-mVenus) were stimulated with increasing concentrations VUF15485 in the absence or presence of increasing concentrations of VUF16840. All values depict the mean ± SD of ≥ 3 experiments.

**Supplemental Table 1 – Ligand potencies to modulate the ACKR3.** The potencies of the inverse agonist VUF16840 and the agonists VUF15485, CXCL11 and CXCL12 to modulate ACKR3 are depicted as pEC_50_ values. The inhibitory potency in which VUF16840 negates the effect of 100 nM of agonists VUF15485, CXCL11 and CXCL12 are depicted as pIC_50_ values. All values depict the mean ± SD of (N) experiments.

| **Mean ± SEM (N)** | | | VUF16840 | VUF15485 | CXCL11 | CXCL12 |
| --- | --- | --- | --- | --- | --- | --- |
| **β-arrestin2** | BRET | pEC_50_ | 8.0 ± 0.1 (7)* | 7.9 ± 0.1 (7) | 8.2 ± 0.1 (3) | 8.5 ± 0.1 (6) |
|  |  | Inhibition by VUF16840 (pIC_50_) | NA | 8.0 ± 0.3 (6) | 8.7 ± 0.2 (3) | 8.6 ± 0.2 (3) |
|  | NanoBit | pEC_50_ | 7.8 ± 0.1 (8)* | 7.8 ± 0.1 (7) |  |  |
|  |  | Inhibition by VUF16840 (pIC_50_) | NA | 7.7 ± 0.0 (3) |  |  |
| **β-arrestin1** | BRET | pEC_50_ | 8.3 ± 0.3 (6)* | 7.4 ± 0.2 (6) | 8.1 ± 0.1 (6) | 8.0 ± 0.1 (5) |
|  |  | Inhibition by VUF16840 (pIC_50_) | NA | 8.7 ± 0.3 (4) | 8.6 ± 0.2 (4) | 8.3 ± 0.1 (4) |
| **Rab5a** | BRET | pEC_50_ | 8.8 ± 0.3 (5)* | 8.1 ± 0.2 (4) |  |  |
|  |  | Inhibition by VUF16840 (pIC_50_) | NA | 8.3 ± 0.2 (4) |  |  |
| **GRK2** | BRET | pEC_50_ | 8.5 ± 0.3 (4)* | 7.6 ± 0.1 (3) |  |  |
|  |  | Inhibition by VUF16840 (pIC_50_) | NA | 8.4 ± 0.1 (3) |  |  |
| **Clathrin A/**  **β-arrestin2 binding** | BRET | pEC_50_ | 8.0 ± 0.3 (5)* | 8.2 ± 0.4 (5) |  |  |
|  |  | Inhibition by VUF16840 (pIC_50_) | NA | 7.3 ± 0.2 (3) |  |  |

*pEC_50_ value for VUF16840 as inverse agonist

**SI References**

1. Schäfer, G; Fleischer, T; Ahmetovic, M; Guerry, P; Abele, S. Development of a Scalable, Racemic First-Generation Route for CXCR7 Antagonist ACT-1004-1239 via *cis*-to-*trans* Epimerization and Subsequent Separation of Enantiomers. *Organic Process Research & Development* **2024** *28* (6), 2090-2102.

2. Schäfer, G; Fleischer, T; Merot, A; Erhardt, M; Mathys, B. Development of a Scalable, Stereoselective Second-Generation Route for CXCR7 Antagonist ACT-1004-1239 via Chiral Enamine Reduction and a Novel Telescoped Sequence of Transesterification, *cis*-to-*trans* Epimerization, and Saponification. *Organic Process Research & Development* **2024** *28* (6), 2103-2116
